# Supplementary material for: Refinement of post-COVID condition core symptoms, subtypes, determinants, and health impacts: a cohort study integrating real-world data and patient-reported outcomes
Source: eBioMedicine. 2024 Dec 10;111:105493. doi: 10.1016/j.ebiom.2024.105493 (PMC11697707; doi:10.1016/j.ebiom.2024.105493)
Supplement: Supplementary Figs. S1–S6 and Table S1 [file mmc1.docx]

**Refinement of post-COVID condition core symptoms, subtypes, determinants, and health impacts: A cohort study integrating real-world data and patient-reported outcomes**

Yunhe Wang^1^, Marta Alcalde-Herraiz^2^, Kim López Güell^2^, Li Chen^2,3^, Lourdes Mateu^4-7^, Chunxiao Li^8^, Raghib Ali^8^, Nicholas Wareham^8^, Roger Paredes^5,9^, Daniel Prieto-Alhambra^2,10*^, Junqing Xie^2^

1: Nuffield Department of Population Health, University of Oxford, Oxford, UK

2: Centre for Statistics in Medicine and NIHR Biomedical Research Centre Oxford, NDORMS, University of Oxford, Oxford, UK

3: Institute of Child and Adolescent Health, School of Public Health, Peking University

4: Department of Infectious Diseases & irsiCaixa AIDS Research Institute, Hospital Universitari Germans Trias i Pujol, Badalona, Catalonia, Spain

5: Chair in Infectious Diseases and Immunity, Center for Health and Social Care Research (CEESS), Faculty of Medicine. University of Vic- Central University of Catalonia (UVic-UCC)

6: Universitat Autònoma de Barcelona, Catalonia, Spain

7: REICOP (Red de Investigación Covid Persistente), Madrid, Spain

8: Medical Research Council Epidemiology Unit, University of Cambridge, Cambridge, UK

9: Center for Global Health and Diseases, Department of Pathology, Case Western Reserve University School of Medicine, Cleveland, OH

10: Department of Medical Informatics, Erasmus University Medical Center, Rotterdam, the Netherlands

*Corresponding to:

Prof. Daniel Prieto-Alhambra

Email: [daniel.prietoalhambra@ndorms.ox.ac.uk](mailto:daniel.prietoalhambra@ndorms.ox.ac.uk)

Centre for Statistics in Medicine and NIHR Biomedical Research Centre Oxford, NDORMS, University of Oxford, Oxford, UK

**Background**

Post-COVID-19 condition (PCC) affects millions of people, and is an essential component of the long-term impact of COVID-19 during the post-pandemic era. Yet, consensus on clinical case definition and core components of PCC remains lacking, affecting our ability to inform research and evidence-based management. Our study aims 1) to identify the most specific symptoms for PCC , and identify clinical subtypes; 2) to evaluate both virus- and host-related determinants of PCC, and 3) assess the impact of PCC on physical and mental health.

**Methods**

We studied participants from UK Biobank who completed a health and wellbeing survey between June and September 2022. Participants reported the current conditions of the presence, duration, and functional limitations of 45 symptoms, using an online questionnaire designed specifically for COVID-19 research. SARS-CoV-2 infection status and disease history were obtained through linkage to surveillance data and electronic medical records, respectively. Participants reporting symptoms within 30 days after infection (acute phase) were excluded. The most specific PCC symptoms were defined using two criteria: statistical significance (P < 0.05 after Bonferroni correction) and clinical relevance (absolute risk increase >5%). Propensity score weighting was used to control for confounding. Subtypes of PCC were then defined based on the specific symptoms among the COVID-19 infected individuals. A multivariable regression was used to study pathogen- and host-related risk factors for PCC, and its impact on 13 physical and 4 mental health patient-reported functional outcomes.

**Findings**

172,303 participants (mean age 68.9, 57.4% female) were included in the analysis, of whom 43,395 had PCR-confirmed COVID-19. We identified 10 most specific symptoms and classified four PCC subtypes: ENT subtype (30.1%), characterized by alterations in smell, taste, and hearing loss; cardiopulmonary subtype (10.4%), characterized by shortness of breath, postural tachycardia, chest tightness, and chest pressure; neurological subtype (23.5%), characterized by brain fog and difficulty speaking; and general fatigue subtype (38.0%), characterized by mild fatigue. A higher PCC risk was observed for patients with Wild-type variant, multiple infections, and severe acute COVID-19 illness, consistently across the four PCC subtypes. In addition, a range of factors, including socioeconomic deprivation, higher BMI, unhealthy lifestyle, and multiple chronic health conditions, were associated with increased PCC risk, except for age and sex. Conversely, vaccination was associated with a largely reduced PCC risk, particularly for the cardiopulmonary subtypes. Individuals with PCC experienced a much worse physical and mental health. Specifically, the cardiopulmonary subtype had the most pronounced adverse impact on function impairments, followed by neurological, mild fatigue, and ENT subtype. The most affected functions included the ability to concentrate, participate in day-to-day work, and emotional vulnerability to health problems.

**Interpretation**

PCC can be categorized into four distinct subtypes based on ten core symptoms. These subtypes appeared to share a majority of pathogen and host-related risk factors, but their impact on health varied markedly by subtype. Our findings could help refine current guidelines for precise PCC diagnosis and progression, enhance the identification of PCC subgroups for targeted research, and inform evidence-based policy making to tackle this new and debilitating condition.

**Research in context**

**Evidence before this study**

We searched PubMed and MEDLINE for articles published between March 1, 2020, and July 1, 2024, using the search terms “post-COVID condition”, “long COVID”, “post-acute sequelae”, “definition”, “subtype”, “cluster”, “risk factor”, “prevention”, “management”, and “treatment”, with no language restrictions. Previous studies with variable designs have reported substantially divergent PCC prevalence and *symptomatology*. Although a consensus-based core symptom set has been developed, the use and reporting of PCC are largely heterogeneous across major organizational *definitions* from WHO, UK NICE, and US CDC, and across real-world studies. In addition, most PCC studies used electronic health records to curate PCC phenotypes, which are known to be limited by heterogeneity in clinical coding, inadequacy and incompleteness of diagnostic codes for the capturing of symptoms, and biases in participant selection and health care use. Furthermore, previous evidence supported that PCC should be regarded as an umbrella condition with multiple *subtypes*, and each may potentially link to distinct physiopathology mechanisms. However, previous studies have identified symptom clusters/ subtypes that span almost all organ and system domains, which were rarely replicable across cohorts. Regarding the *determinants*, although PCC has been associated with factors such as age, sex, and ethnicity, whether a range of host- and virus-related determinates of overall PCC also consistently related to various PCC subtypes remains unclear. The impact of PCC and its subtypes on patient-reported *health and functioning* are also still not widely recognised. These knowledge gaps in the condition core symptoms, subtypes, determinants, and health impacts of PCC should be addressed to inform evidence-based policy making to tackle this new and debilitating condition.

**Added value of this study**

In this study integrating both longitudinal electronic medical records and patient-centred data among a large community-based cohort of 172,303 participants, we discerned the ten most indicative symptoms for the diagnosis of PCC. Based on the identified symptomatology, we classified PCC into four major subtypes, dominated by ENT, respiratory and cardiac symptoms, cognitive impairments, and mild fatigue. In addition, we provided an in-depth evaluation of the determinants of each PCC subtype, including both virus- and host-related aspects: infection by pre-omicron variants, repeat infection, severe acute COVID-19, overweight and obesity, unhealthy lifestyle, and multiple comorbidities significantly increased PCC risk across all subtypes. Conversely, vaccination against COVID-19 (primary and booster) significantly reduced the risk of all PCC subtypes. Pre-existing comorbidities were more closely associated with PCC symptoms affecting the same organs/systems. For example, COPD and depression led to higher risks of cardiopulmonary and neurological subtypes, respectively. Lastly, our results reveal that the cardiopulmonary and neurological subtype had the strongest adverse impacts on both physical and mental health. These results highlight the heterogeneity in the presentation of PCC, demographic and clinical risk factors, and related functional impact of PCC, and the importance of characterizing the underlying mechanisms. These novel findings have great potential to advance the current understanding, diagnosis, and management of the long-term adverse effects of COVID-19 during the post-pandemic era.

**Implications of all the available evidence**

By combining the EHR and patient-reported data, our investigation showed that PCC can be categorized into four distinct subtypes based on ten core symptoms. These subtypes appeared to share a majority of pathogen (e.g., COVID-19 variants, multiple infections, hospitalization, and vaccination) and host-related (age, sex, healthy lifestyle, BMI, socioeconomic factors, and multiple comorbidities) risk factors, but their impact on health varied markedly by subtype. Our findings could help refine current guidelines for precise PCC diagnosis and progression, enhance the identification of PCC subgroups for targeted research, and inform evidence-based policy making to tackle this new and debilitating condition.

# **Introduction**

*Post-COVID-19 conditions (PCC)* or *long COVID*, generally defined as ongoing, relapsing, or new onset multisystem symptoms or related conditions during the post-acute stage of SARS-CoV-2 infection, constitute a global public health crisis. This novel clinical entity can persist up to 3 years following the initial infection,^1,2^ which has affected millions of people worldwide,^3^ and had significant impacts on daily functioning, quality of life, work, and healthcare costs.^4^ Unfortunately, limited management strategies have been established to date. There is therefore an urgent need to research PCC-related symptomatology and determinants to inform effective prevention and treatment strategies.

Previous studies with variable designs have reported substantially divergent PCC prevalence and symptoms.^5^ The lack of consensus on case definition and core components of PCC has also led to heterogenous outcome measures,^6^ undermining the comparability between studies and limiting our ability to inform evidence-based management. The four key elements for precision PCC diagnosis should include a core symptom set,^7^ timeframe from infection, persistence, and impacts on daily functioning. However, the adoption of these elements varied significantly across major organizational definitions from WHO, UK NICE, and the US CDC,^8^ and across many previous studies.^5^ In addition, most PCC studies used electronic health records (EHRs) to curate PCC phenotypes, which are known to be limited by heterogeneity in clinical coding, inadequacy and incompleteness of diagnostic codes for the capturing of symptoms, and biases in participant selection and health care use.^9^

PCC should be regarded as an umbrella condition with multiple subtypes, and each may link to distinct physiopathology mechanisms.^10-14^ Yet, previous studies have identified symptom classes that span almost all organ and system domains, which were rarely replicable across cohorts. Notably, although PCC has been associated with factors such as age, sex, and ethnicity,^10,11,14^ whether a range of host- and virus-related determinates of overall PCC also consistently related to various PCC subtypes remains unclear. Furthermore, the impact of PCC and its subtypes on patient-reported health and function are still not widely recognised. Accurate refinement of PCC symptomatology, determinants, and health impacts can potentially help reveal the pathophysiology of PCC and inform targeted care strategies.^15^

To fill these knowledge gaps, we used comprehensive data with detailed patient-centred outcomes from participants in a large and rich prospective population-based cohort, and set out to: (1) identify the most specific symptoms (MSS) of PCC and its clinical subtypes; (2) characterize the virus- and host-related determinants of PCC; and (3) assess the impact of PCC on physical and mental health and functioning.

# **Methods**

**Data sources**

We used data from the UK Biobank, a prospective population-based cohort study with over 500,000 participants recruited between 2006 to 2010 across England, Scotland, and Wales. Besides the comprehensive baseline data, several external databases have been linked to UK Biobank participants to enable the follow-up of their health and disease outcomes.^16,17^ Details of the cohort profile and data linkages have been described previously.^18^

During the COVID-19 pandemic, an online Health and Well-being survey was conducted for all survivals of UK participants in order to assess the impact of COVID-19 by directly collecting data from patients. Initially, a pilot study involving 10,000 participants was first conducted to ascertain the robustness of the survey platform and the acceptability of the questionnaire's content and length. Subsequently, 333,427 individuals were invited via email, and 60.5% (201,684) completed the online questionnaire between 22^nd^ June 2022 and 15^th^ May 2023. A further 1,094 participants without email invitation completed the survey directly via the UK Biobank website. The questionnaire included questions on current health issues, mental health conditions, and daily functional impairments, designed by an expert panel with expertise in long COVID. It incorporated items from the Wellcome COVID-19 questionnaire and surveys from other extensive cohort studies such as REACT, TwinsUK, and the ZOE COVID Study. Consultation with the researchers who developed a CORE outcome set for Long COVID was also involved to ensure the inclusion of symptoms most relevant to post-acute COVID. **Supplementary Table 1** lists the origins and sources of questionnaire items. Details of Health and Well-being survey are provided online (https://biobank.ctsu.ox.ac.uk/crystal/refer.cgi?id=2500).

**Study design**

We performed a retrospective cohort and a cross-sectional analysis among UK Biobank individuals who had completed the online Health and Well-being survey.

For the cohort analysis of PCC symptoms, we curated an COVID-19 infection group who are participants with a prior PCR-confirmed SARS-CoV-2 infection, and a control group who are participants without the infection. PCR-based testing results for SARS-CoV-2 were obtained from Public Health England’s Second Generation Surveillance System (PHE-SGSS), a centralized microbiology database covering English clinical diagnostics laboratories that had been previously validated for COVID-19 research.^19,20^ The infection status was confirmed before or on the date of the survey when the patient-reported outcomes were collected (*T_s_*). Specifically, in the infected group, we defined the infection date as the index date (*T_0_*) when the follow-up starts. For those with multiple infections, date of the most recent infection was used as the *T_0_* , given that the PCC being studied may be more closely associated with recent than earlier ones. Whereas in the control group, we assigned a random *T_0_* based on the calendar time distribution in the infection group, which ensures an comparable follow-up duration (*T_s_*-*T_0_*) between the two groups. Subsequently, we excluded people who completed the survey within 30 days of the infection, to ensure the reported symptomatology related to post-acute COVID only. Overall, 3% of remaining participants were excluded due to failure of the initial identity check. Based on the identified PCC symptomatology, we classified PCC as those who reported at least one PCC core symptoms during the survey and had a record of COVID-19 30 days before the survey date.

For the cohort analysis of PCC risk factors, COVID-19 group, and two reference groups were defined: (1) participants reported no PCC core symptom but previously infected with COVID-19, and (2) participants previously not infected with COVID-19. For pathogen-related risk factors, we compared the COVID-19 group with uninfected participants. For host-related risk factors, we compared the PCC group with those without PCC among the COVID-19 group.

For the cross-sectional analysis of PCC’s health impact, we compared the PCC group with those without PCC among the COVID-19 group.

A study design diagram for both analyses is depicted in **Supplementary Figure 1**.

**Covariates**

We prespecified covariates previously related to COVID-19 for the adjustment of potential confounders based on previous research,^19,20^ including age, sex, index of multiple deprivation (IMD), education, ethnicity, body mass index, smoking status, physical activity level measured by the International Physical Activity Questionnaire, and all prior comorbidities with prevalence larger than 1% in our cohort.

**Outcomes and measurements**

The primary outcomes of the current study were the presence of 45 prespecified somatic and mental symptoms potentially related to COVID-19. This information was collected in the questionnaire based on participants' responses to the question: "Are you currently suffering from any of the following health issues that are new or have worsened in recent weeks?". For those reporting symptoms, their impact on daily life and activities was further investigated through the question: "To what extent is the issue affecting you?", with answer options of ‘It is not limiting me’ and ‘It is causing me to avoid, reduce or spread out my usual activities’. The correlation between these 45 symptoms in 7 domains is provided in **Supplementary Figure 2**.

Secondary outcomes included a predefined list of mental and physical function impairments, identified through the question: “Over the last 2 weeks, how often have you been bothered by any of the following problems?” for mental health using PHQ-4, and “How much difficulty do you have with the following activities?” for physical functioning. Response options were structured on a four-point Likert scale for the former question ranging from ‘Not at all’, ‘Several days’, ‘More than half the days’ and ‘Nearly every day’, and a five-point Likert scale for the later question ranging from 'No difficulty', 'Mild difficulty', 'Moderate difficulty', 'Severe difficulty', to 'Extreme difficulty'.

We defined answers of ‘Do not know’ or ‘Prefer not to answer’ as missing values. Given that few data were missing, with the highest proportion of missing values observed for the symptoms of hearing loss (2.9%) and bone pain (2.8%), we thus conducted the complete case analysis for this study.

**Statistical analysis**

We used propensity score (PS) weighting to control for difference in baseline characteristics between the COVID-19 infected group and the control group. We built a multivariable logistic regression with Lasso L1 penalty to estimate the PS as the probability of belonging to the infection group, using all covariates listed above. Inverse probability weights were calculated as one divided by the PS in the infection group and divided by one minus PS in the control group. We then examined the covariate balance between groups. An absolute standardized mean difference (SMD) less than 0.1 were regarded as well balanced. The same PS weighting procedures were performed both in the longitudinal cohort and cross-sectional analysis.

We used PS-weighted logistic regression to calculate odds ratio (OR) for the primary and secondary outcomes. We used the most strict Bonferroni method^21^ to correct for multiple testing in our study and reported corrected confidence intervals under the P < .05/(45) for the primary outcomes and P < .05/(17) for the secondary outcomes. The most specific symptoms (MSS) of PCC were identified using a dual criteria approach^22^: statistical significance (*P* < 0.05 after Bonferroni correction) and clinical relevance (relative risk increase >5%), after the propensity score adjustment. PCC was defined as the presence of at least one MSS symptom ≥30 days after a COVID-19 infection in the main analysis. PCC subtypes were further classified as the presence of symptoms in each relevant domain, according to existing literature, clinical knowledge, and expert opinion.

We assessed potential determinants for PCC, including pathogen-related factors (SARS-CoV-2 variants, reinfection, hospitalization due to COVID-19) and host-related factors (age, sex, IMD, ethnicity, obesity, lifestyle factor, comorbidity, and COVID-19 vaccination status). SARS-CoV-2 variants were classified according to the calendar period of predominant strain reported in the UK (Wild: 01/03/2020 to 31/12/2020, Alpha: 01/01/2021 to 31/07/2021, Delta: 01/08/2021 to 30/11/2021, Omicron: after 01/21/2021). Lastly, we assessed the impact of PCC on daily functioning based on 13 physical and 4 mental variables.

We conducted two sensitivity analyses. First, we excluded participants who had an infection within 90 (instead of 30) days prior to the survey to define PCC and its subtypes. The adjustment was made as there is no uniform definition for long COVID, which is currently described as conditions occurring 30-90 days after infection in existing guidelines. Second, we defined PCC and its subtypes as the presence of specific symptoms developed 90 days following SARS-CoV-2 infection and last for less than twelve weeks with impact on daily functioning, which is largely consistent with the current WHO definition of long COVID.

# **Results**

**Baseline characteristics of cohorts**

Study design and the process of cohort construction are provided in Supplementary **Figure 1**. Out of 195,680 respondents to the Health and Well-being survey in the UK Biobank, 23,357 individuals were excluded due to either completing the questionnaire after the end date of data linkage (September 30, 2022; 90% of participants completed the survey by August 30, 2022) or having COVID-19 within 30 days prior to the symptom reporting date. Of these, 43,395 were identified with PCR-confirmed COVID-19, constituting the infection cohort, while the remaining 128,908 served as *uninfected controls*. **Supplementary Figure 3** illustrates the distribution of index dates for the infection and control cohorts over the study period (March 2020-Augest 2022). Both groups showed a similar median interval between the index and symptoms reporting dates (215 days in the infection and 218 days in the control). Before weighting, the absolute SMDs for all covariates were below 0.1, except for age: participants in the control group were approximately 2 years older compared with the infection group (mean [SD] age: 69.5 [7.5] years vs 67.3 [7.7] years, SMD). All other socio-demographic factors and comorbidities were similar between groups. Prevalence of chronic diseases were similar between groups. After PS weighting, all characteristics were well balanced (SMD <0.1), and index dates were fully aligned (**Table 1** and **Supplementary Figure 3**).

**Specific symptoms and PCC subtypes**

In PS-weighted cohorts, 17 of 45 symptoms showed positive and statistically significant associations with COVID-19 (BF adjusted P-value <0.05) (**Figure 1**). Among these, seven symptoms did not meet the clinical significance threshold, with an OR <1.05: "tinnitus", "other hearing issues", "heart issues", "joint pain or swelling of joints", "night sweats", "unrestful sleep", and "difficulty sleeping". The clinically significant ten symptoms, ranked by effect sizes, were: "loss or change in sense of smell", "loss or change in sense of taste", "hearing loss", "shortness of breath or trouble breathing", "postural tachycardia", "tightness in the chest", "chest pressure/heaviness", "problems thinking (e.g., brain fog)", "problems communicating (e.g., difficulty speaking)", and "mild fatigue". Notably, eight symptoms showed inverse and significant associations with COVID-19 (OR<0.95): "nasal congestion", "sore or painful throat", "persistent cough", "phlegm production or chesty cough", "nausea and/or vomiting", "decrease in appetite", "fever (feeling too hot)", and "chills (feeling too cold)".

Based on the ten specific symptoms associated with COVID-19, we further ascertained four PCC subtypes: ENT subtype (characterized by alteration in sense of smell and taste and hearing loss), cardiopulmonary subtype (characterized by shortness of breath, postural tachycardia, and chest tightness/pressure), neurological subtype (characterized by brain fog and difficulty speaking), and mild fatigue subtype (characterized by mild fatigue). Overall, 60.1% of participants in the infection group and 57.4% *uninfected controls* had at least one of the ten specific symptoms. The prevalence of PCC subtypes in the infection group was (in order): 38.0% for mild fatigue, 30.1% for ENT, 23.5% for neurological, and 10.4% for cardiopulmonary subtype. These subtypes were also common in the uninfected control group: 36.3% for mild fatigue, 28.0% for ENT, 21.4% for neurological, and 9.52% for cardiopulmonary subtype.

**Pathogen-related factors**

The effects of pathogen-related factors with PCC were generally consistent across PCC subtypes (**Figure 2**). The association between COVID-19 and PCC showed great variation across the SARS-CoV-2 variants. The risk for PCC and its subtypes consistently decreased from the initial Wildtype (OR for any PCC 1.32 [95% CI 1.26 to 1.38]) through Alpha and Delta to the most recent Omicron variant (1.07 [1.06 to 1.09]). The only exception was for the ENT subtype, where the Delta variant showed the highest risk (Delta 1.36 [1.32 to 1.41] vs Wildtype 1.27 [1.21 to 1.33]). Participants with multiple infections (1.41 [1.33 to 1.50]) had a higher risk of any PCC compared to those with a single infection (1.10 [1.09 to 1.12]). Similarly, those hospitalized due to COVID-19 within 30 days post-infection (OR 2.01 [95% 1.09 to 2.12]) showed a consistently increased risk of PCC compared to non-hospitalized participants (1.09 [1.07 to 1.11]). These associations were consistently observed across PCC subtypes. Full vaccination and booster were associated with significantly lower risk of PCC compared to no or partial vaccination (no/partial 1.25 [1.20 to 1.29] vs booster 1.08 [1.06 to 1.10]). Risk of ENT subtype was higher in participants with full vaccination, coinciding with the dominance of the Delta variant during the period of increasing vaccine uptake (data not shown). Notably, wildtype infections, multiple infections, and hospitalization due to COVID-19 were more strongly associated with the cardiopulmonary PCC subtype, suggesting that the number and severity of acute SARS-CoV-2 infections may be more directly and biologically related to post-acute cardiopulmonary symptoms than multiple extrapulmonary sequelae.

**Host-related factors**

A range of sociodemographic and clinical factors were significantly associated with the risk of PCC, with similar effects observed across PCC subtypes for most factors except for age and sex (**Figure 3**).

Compared with those aged 45-55 years, participants aged 55-65 (OR 0.79 [0.71 to 0.88]) and 65-75 (0.79 [0.70 to 0.88]) years were at a lower risk of any PCC, while those over 75 years were at increased risk (1.23 [1.09 to 1.39]). However, the age patterns varied across PCC subtypes. The risk of ENT subtype increased with a gradient of increasing age, whereas participants with neurological and mild fatigue subtypes were more likely to be younger. Females were at increased risk for PCC overall (OR 1.29 [1.24 to 1.35]), and for cardiopulmonary, neurological, and mild fatigue subtypes, but at lower risk of ENT subtype (0.88 [0.84 to 0.93]). Overall, PCC risk increased with socioeconomic deprivation, with an OR of 1.20 [1.14 to 1.28] for those living in the most deprived areas. No difference in risk of PCC were seen across ethnic groups, except for the ENT subtype.

The risk for PCC overall and across subtypes increased monotonically with BMI, with OR ranging from 1.50 (95% CI 1.32 to 1.71) for ENT to 2.29 (1.94 to 2.70) for cardiopulmonary subtypes in those with BMI ≥35 compared to BMI <25. Adherence to a healthy lifestyle was consistently associated with lower risk of PCC overall (OR 0.63 [0.58 to 0.67]) and subtypes. Vaccination was associated with a significant reduction in the risk of PCC, overall and by subtype.

All studied comorbidities showed a positive association with PCC (overall and subtypes), except for chronic kidney disease in relation to neurological PCC (OR 0.99 [0.83 to 1.17]). COPD and depression appeared to be the most significant comorbidities related to the risk of PCC (OR 2.05 [1.72 to 2.44] and 1.89 [1.75 to 2.04], respectively), with COPD having the strongest effect on cardiopulmonary subtype (5.04 [4.12 to 6.16]) and depression on neurological subtype (2.24 [2.08 to 2.40]). The prevalence of comorbidities by PCC status is provided in **Supplementary Figure 4**.

**Impact on physical and mental health and functioning**

Individuals with PCC experienced a marked impact on all measured physical health/function metrics, although the magnitude of associations varied considerably across PCC subtypes and outcomes (**Figure 4a**). The ENT subtype was generally associated with the smallest impact on physical function (highest OR: 6.42 [5.97 to 6.90] for “doing day to day work”), followed by mild fatigue subtype (highest OR 9.35 [8.48 to 10.31] for “doing day to day work”), and cardiopulmonary subtype (highest OR 14.71 [13.64 to 15.87] for “doing day to day work”). Notably, the neurological subtype had the strongest adverse impact (highest OR: 16.58 [15.11 to 18.19] for “concentrating on doing something for ten minutes” among 13 difficulties).

Across these physical outcomes, the “being emotionally affected by health problems”, “taking care of household responsibilities”, “doing day to day work”, and “concentrating on doing something for ten minutes” were the four physical functions most adversely affected by PCC and its subtypes.

People with PCC also experienced a great impact on mental health and functioning (**Figure 5b**). Although the associations with mental health appeared to be less pronounced than with physical functions, the patterns of association remained similar across PCC subtypes. For instance, the ENT subtype generally showed the lowest OR across mental functioning outcomes, with the neurological subtype had the strongest impacts.

Generally, a higher number of coexisting PCC subtypes led to worse impacts on physical and mental health. Adjusted ORs for physical outcomes ranged from 1.83 [1.70 to 1.96] to 3.04 [2.82 to 3.27] in those with two concurrent PCC subtypes, and from 10.78 [9.77 to 11.90] to 40.58 [36.80 to 44.76] in those with four concomitant subtypes. Similarly, OR of mental outcomes ranged from 1.96 [1.88 to 2.05] to 2.70 [2.53 to 2.88] in those coexisting with two subtypes, and from 10.59 [9.77 to 11.48] to 23.73 [21.69 to 25.95] in those with four subtypes.

Sensitivity analyses using definitions based on 90-day windows identified broadly similar symptomatology patterns (**Supplementary Figure 5**). Prevalence of 45 PCC-related symptoms in participants with and without COVID-19 by definition are provided in **Supplementary Figure 6**.

# **Discussion**

In this comprehensive study integrating both longitudinal electronic medical records and patient-centred data among a large community-based cohort, we discerned the ten most indicative symptoms for the diagnosis of PCC. Based on the identified symptomatology, we classified PCC into four major subtypes, dominated by ENT, respiratory and cardiac symptoms, cognitive impairments, and mild fatigue. In addition, we provided an in-depth evaluation of the determinants of each PCC subtype, including both virus- and host-related aspects: infection by pre-omicron variants, repeat infection, severe acute COVID-19, overweight and obesity, unhealthy lifestyle, and multiple comorbidities significantly increased PCC risk across all subtypes. Conversely, vaccination against COVID-19 (primary and booster) significantly reduced the risk of all PCC subtypes.

Pre-existing comorbidities were more closely associated with PCC symptoms affecting the same organs/systems. For example, COPD and depression led to higher risks of cardiopulmonary and neurological subtypes, respectively. Lastly, our results reveal that the cardiopulmonary and neurological subtype had the strongest adverse impacts on both physical and mental health. These results highlight the heterogeneity in the presentation of PCC, demographic and clinical risk factors, and related functional impact of PCC, and the importance of characterizing the underlying mechanisms. These novel findings have great potential to advance the current understanding, diagnosis, and management of the long-term adverse effects of COVID-19 during the post-pandemic era.

**Main findings in context**

*Symptomatology of PCC*

Despite notable difference in target populations and methodologies used (data source, study design, follow-up length, and analytical approach), the symptomatology patterns across four major PCC subtypes identified in the current study are highly aligned with prior key studies. For example, a cohort study in the Netherlands utilised repetitive cross-sectional design. It proposed 10 core PCC somatic symptoms,^23^ including respiratory-cardiac symptoms, olfactory-auditory symptoms, and general fatigue. Another similar longitudinal study in Switzerland identified 6 symptoms that were at the most excess risk 6 months after infection ("altered taste or smell", "dyspnoea”, "post-exertional malaise", “reduced concentration”, “memory”, and "fatigue").^24^ Also, a population-based study of German participants found four symptom clusters, including fatigue, neurocognitive impairment, chest symptoms, and smell or taste disorders.^25^ Similarly, a UK-based study found the top 7 symptoms at 12 weeks following infection were loss or change of sense of smell or taste, shortness of breath, fatigue, difficulty thinking or concentrating, chest tightness or pain, and poor memory.^26^ Together, these consistent findings suggest the potential utility of the identified MSS for the accurate assessment of patients with PCC in routine clinical practice, and for the effective recruitment of trial participants, warranting further validation across diverse populations and settings.

Differences, however, were also noted. Many studies have associated gastrointestinal symptoms, such as dyspepsia and constipation, and musculoskeletal symptoms, such as joint, muscle or back pains, with COVID-19 following the acute infection.^14,27,28^ Most of these studies were conducted based on routinely collected data from electronic health records (EHRs). There is a growing awareness that capturing the symptomatology through real-world clinical data could be challenging.^9^ Also, due to the nonspecific nature of symptoms, most are often not well-represented by diagnostic coding schemes such as ICD-10. Our study analysed comprehensive self-reported data and found neither one of the gastrointestinal or musculoskeletal symptoms to be significantly associated with PCC, although they tended to be very common after COVID-19 and in those without infection. Feeling hot/cold, an acute COVID-19 symptom, was also suggested as a core PCC symptom,^23^ but it was inversely associated with PCC in our study. Interestingly, the inverse association was consistently observed for other typical acute symptoms, such as sore throat, cough, nausea, and appetite decrease. This phenomenon might be explained by individual’s perception change over time since the infection: infected people could be overly attentive to typical acute symptoms during the early phase of infection, which may in turn result in a significant reduction of reporting the same symptoms once physically recovered or psychologically relieved.^29^ More investigations are needed to clarify this potential self-reporting bias in research focusing on PCC symptoms. Another possible explanation to this could be the misidentification of acute infection cases likely due to Omicron variants in the control group, given that, since 1 April 2022, routine COVID-19 testing was no longer recommended in many UK settings, and free mass testing for the general public was largely phased out.^30^

*Host- and virus-related determinants*

Our study also provides novel insights into the determinants for PCC development. Across PCC subtypes, the effect pattern of virus-related factors appears consistent, yet often varies by host-related factors. The effects of most risk factors across subtypes, such as deprivation, unhealthy lifestyle, obesity, and comorbidities, are consistent with previous findings that mainly do not distinguish PCC subtypes, suggesting the health inequalities in PCC and the potential benefits of adherence to a healthy lifestyle in its prevention. However, heterogenous effects were observed for age and sex. A meta-analysis of 41 studies suggested that older age and female sex are independent risk factors for PCC.^31^ Our findings generally support these associations but revealed that their effects may differ depending on the specific PCC subtype, with younger age related to higher risk of neurological and fatigue subtypes. This variation may partially explain the prior inconsistent evidence in this regard, where data from the UK Office for National Statistics showed that the middle-aged adults have the highest prevalence of PCC,^32^ suggesting that aging might have a two-way effect. Similarly, while females had a higher PCC risk than males, this may not be uniformly applicable to all PCC subtypes, as ENT subtype appears more prevalent in men. Additionally, our findings into specific comorbidities and risk for different PCC subtypes has implications for the management of PCC. Organ-specific comorbidities such as COPD and depression were more closely related to corresponding cardiopulmonary and neurological subtypes, highlighting that baseline organ-level deficits can affect PCC subtype/s risk.

Virus-related factors have relatively consistent effects on PCC and across subtypes. The risk of PCC following infections of different SARS-CoV-2 variant is not yet conclusive, with comparable or lower PCC risk following Omicron infection compared with Delta and other variants.^33,34^ Our study robustly suggested that individuals who first infected during the Omicron-dominated period are at the lowest PCC risk across subtypes, despite the shortest time window since the infection. Nevertheless, the PCC risk remains in individuals following Omicron infection as compared to those uninfected, it is thus foreseeable that the absolute number of new PCC cases would likely to keep accumulating as infections continue. Moreover, our study confirmed further that reinfection and disease severity as important risk factors for PCC^35,36^ and subtypes, especially the cardiopulmonary sequelae. Importantly, people infected after receiving the primary two-dose vaccination were at a significantly lower risk than those who were unvaccinated or only partially vaccinated. However, their risk remained relatively higher than those who had received booster doses. Together, this finding provides further evidence that vaccination may offer protection beyond the acute illness to post-acute complications.^37^ Given that the SARS-CoV-2 variants and vaccination status are two highly correlated conditions, it is hardly to disentangle their individual effects in our study but warrants further research.

*Physical and mental function impairments*

Evidence on the functional impact of PCC was primarily based on survey studies, with high prevalence of functional limitations reported among individuals who self-reported living with PCC.^4^ For example, 78.5% of participants from the US Household Pulse Survey, 58.6% from the UK ONS, and 21.3% from the Canadian national survey experienced daily activity limitations.^4^ However, the lack of a matched control group limited our ability to infer the direct impact of PCC, given that most of these outcomes were also common following COVID-19 and in the general population during the pandemic. Other studies with control groups consisted of participants mainly from post-COVID-19 support groups or used self-diagnosed PCC, undermining the generalizability and validity of findings due to selection and self-reporting biases. After accounting for a comprehensive list of underlying comorbidities, behavioural and sociodemographic factors, we found that PCC and its subtypes were associated with a range of physical and mental health impairments. Overall, the most commonly affected functions were the ability to concentrate, participate in day-to-day work, and take care of household; participants with PCC were also more emotionally vulnerable to health problems and had worse general mental health. Our data suggested that neurological PCC had the most significant adverse impact on physical and mental functions. Although the increased risk of a range of multiorgan symptoms was reported during the 6 to 12 months following SARS-CoV-2 infection,^1^ only the increased risk of cognitive deficit (brain fog) and dementia persisted up to two years.^38^ These findings suggest that service provision and mitigation strategies on the neurological sequelae of COVID-19 should be prioritised and sustained. Severity and subtypes of PCC should be considered when providing targeted care and disability support services.

*PCC subtypes with varying symptomatology, risk factors, and function impairments*

The four major PCC phenotypes identified based on patient-centred data, including the ENT, cardiopulmonary, neurological and general fatigue subtypes, as well as their impairments on physical and mental function, corroborate the current empirically-based consensus for the core outcome set (COS) of PCC.^7^ In the Delphi consensus study by both investigators and patients with lived experience of PCC, the COS included “functioning, symptoms, and conditions” for the cardiovascular, respiratory, nervous system, cognitive, mental health outcomes, and fatigue and pain in the physiological or clinical outcomes domain, and two physical outcomes in the life impact outcomes including work or occupational and study changes and other changes in physical function.^7^ Our study showed consistent symptomatology patterns and function impairments across multiple domains, with the exception of pain. It is likely that the preferred outcomes by patients with lived experience might differ from outcomes considered important by investigators or clinicians,^7^ especially considering that non-specific pain is one of the most common symptoms of PCC.^5^ We confirmed an increased risk of joint pain following post-acute infection, though with low clinical relevance. Further, the phenotypes identified in our study using patient-reported symptom data largely align with symptom clusters in two nationally representative studies.^13,14^ A study of non-hospitalized adults in England^14^ identified three clusters from 50 consolidated symptoms using primary care medical records: class 1 dominated by a range of non-specific symptoms including pain, fatigue and rash (80.0%), class 2 dominated by respiratory symptoms such as cough and shortness of breath (5.8%), and class 3 dominated by neurological and mental health outcomes such as depression, anxiety, insomnia, and brain fog (14.2%). An US study using RECOVER cohort^13^ also found that four clusters featured by olfactory dysfunction, cardiopulmonary sequelae, neurocognitive impairment, and fatigue. Similarly, these findings do not support the musculoskeletal and digestive symptom clusters previously prioritised in most EHR-based studies,^11,12^ although similar cardiopulmonary and neurological subtypes were also identified in these studies.

The effects of socioeconomic and clinical risk factors were largely consistent across the symptom clusters, however, varying effects of demographics such as age, sex, and ethnicity were observed in the current and previous studies.^10,11,14^ Distinct gene expression patterns during acute infection and inflammation signatures were linked to different symptom clusters, highlighting the heterogeneous etiologies of PCC subtypes.^39,40^ In light of our findings and previous evidence, heterogenous phenotypes of PCC with varying distinct patterns of symptoms, risk factors, and related burden of function impairments need to be considered in the definitions, diagnosis, and management of PCC; there is also the need to better understand underlying mechanisms linked to various phenotypic subtypes and develop targeted therapies for different subtypes using precision medicine to improve outcomes for people living with PCC.

**Strengths and limitations**

The major strengths of this study are the size and prospective nature of our community-based cohort, and the richness of information available on longitudinal health, comorbidities, and on self-reported symptoms. The bespoke study design not only allowed for a prospective comparison between COVID-19 patients and concomitant uninfected controls enrolled from the same source population, but also a thorough adjustment for multiple covariates. This thus enabled us to minimise confounding, an issue frequently seen in prior EHR-based studies on this topic. Moreover, all the assessed PCC symptoms were pre-determined based on a consensus from an expert panel, and the resulted questionnaire has been validated. Lastly, the integration of multi-source clinical data with patient-reported outcomes enables the research of different risk factors and consequences of PCC within a large-scale cohort. These methodological features collectively strengthen the validity of our study findings.

Our study findings should be interpreted with caution in the context of some limitations. First, UK Biobank participants are likely to be older and healthier than the UK general population, and are mostly White European, which may limit the generalizability of study findings. Despite the relative risk between comparison groups was largely not influenced,^41^ the absolute estimate of symptomatology prevalence should be interpreted with caution. Second, despite robust statistical approach and a range of covariates were used to account for potential differences in characteristics between groups, residual confounding, measurement error, selection bias, recall bias, and reverse causality cannot be totally ruled out in this observational study. Third, a proportion of participants in the uninfected control group may have undiagnosed or untested COVID-19. However, this tends to underestimate the effect size and leads to more conservative results of symptom selection. Fourth, the molecular mechanisms driving the distinct PCC subtypes should be further explored in future studies with bespoke design. Fifth, debate persists in the literature regarding the optimal use of PS, such as residual confounding or over-reliance on covariate selection criteria, which should be taken into consideration when interpreting the associations observed as causal. Finally, the selection of symptoms most specific to long COVID from a prior consensus symptom list was driven by both statistical significance and clinical relevance. However, it is likely that several unselected symptoms such as general pain may also be more commonly reported in patients with PCC if the baseline risk factors such as comorbidities influence the likelihood of healthcare use and symptom reporting. Further guidelines are needed to integrate evidence from patients, investigators, and clinicians for better management of PCC phenotypes and their impacts.

# **Conclusions**

We identified four PCC subtypes with distinct symptomatology, functional impact, and some differential risk factors. PCC affected mental and physical health and functions significantly. Our findings will guide future assessment, management, and research on PCC as we aim to better understand distinct mechanisms linked to PCC; and ultimately to develop targeted therapies to improve the patient outcomes.

**Acknowledgements**

This study was based on data from UK Biobank. All participants provided written informed consent at the UK Biobank cohort recruitment. This study received ethical approval from UK Biobank Ethics Advisory Committee (EAC) and was performed under the application of 65397. Mr Wang is funded through the Clarendon Fund Scholarship. Dr Xie is funded through Jardine-Oxford Graduate Scholarship and a titular Clarendon Fund Scholarship. The research was partially supported by the Oxford National Institute for Health and Care Research (NIHR) Biomedical Research Centre. Dr Prieto-Alhambra is funded through an NIHR Senior Research Fellowship (grant SRF-2018-11-ST2-004). The funding organizations had no role in the design and conduct of the study; collection, management, analysis, and interpretation of the data; preparation, review, or approval of the manuscript; and decision to submit the manuscript for publication. The views expressed in this publication are those of the author(s) and not necessarily those of the NHS, the NIHR or the Department of Health.

**Data Sharing Statement**

Researchers can apply to use the UK Biobank dataset by registering and applying at https://ukbiobank.ac.uk/register-apply/. Any additional summary data generated and/or analysed during the current study are available from the corresponding author on reasonable request.

**Author contributions**

Y.W and J.X. had full access to all the data in the study and take responsibility for the integrity of the data and the accuracy of the data analyses. Y.W., J.X. and D.P.-A. conceptualized and designed the study, and acquired, analyzed or interpreted data. Y.W. and J.X. drafted the manuscript. J.X., Y.W. and M.H conducted statistical analysis. D.P.-A. obtained funding, provided administrative, technical or material support. R.A, N.W and D.P.-A. supervised the project. All authors critically reviewed/revised the manuscript for important intellectual content.

**Competing interests**

D.P.-A. reported grants from Amgen, UCB Biopharma, Les Laboratoires Servier, Novartis, and Chiesi-Taylor, as well as speaker fees and advisory board membership with AstraZeneca and Johnson and Johnson outside the submitted work, in addition to research support from Janssen. R.P. has participated in advisory boards for Gilead, MSD, ViiV Healthcare, Theratechnologies and Lilly. His institution has received research support from Gilead, MSD, and ViiV Healthcare. The remaining authors declare no competing interests.

**Figure legend**

**Figure 1. Associations of COVID-19 with the presence of 45 symptoms potentially related to PCC during the post-acute phase of infection.**

**Figure 2. Associations of SARS-CoV-2 pathogen-related factors with PCC and its subtypes.**

**Figure 3. Associations of host-related factors with PCC and its subtypes.**

**Figure 4. Associations of PCC and its subtypes with physical and mental function impairments.**

**Table 1. Baseline characteristics of COVID-19 infection vs uninfected control group before and after propensity score weighting**

|  | **Before weighting** | | |  | **After weighting** | | |
| --- | --- | --- | --- | --- | --- | --- | --- |
| **Characteristics** | **Control** | **Infection** | **SMD** |  | **Control** | **Infection** | **SMD** |
| Number | 128908 | 43395 |  |  | 172203 | 171390 |  |
| Age (years, SD) | 69.48 (7.49) | 67.27 (7.65) | 0.291 |  | 68.93 (7.60) | 68.87 (7.55) | 0.009 |
| Sex (Male, %) | 54872 (42.6) | 18644 (43.0) | 0.008 |  | 73400.0 (42.6) | 73980.8 (43.2) | 0.011 |
| Ethnicity (White, %) | 118629 (92.0) | 40020 (92.2) | 0.007 |  | 158477.0 (92.0) | 157950.2 (92.2) | 0.005 |
| Socioeconomic deprivation (n, %)* |  |  | 0.041 |  |  |  | 0.019 |
| Q1 (least deprived) | 32711 (25.4) | 10365 (23.9) |  |  | 43377.2 (25.2) | 42015.2 (24.5) |  |
| Q2 | 32340 (25.1) | 10736 (24.7) |  |  | 43004.1 (25.0) | 43037.1 (25.1) |  |
| Q3 | 32008 (24.8) | 11068 (25.5) |  |  | 42781.0 (24.8) | 43644.7 (25.5) |  |
| Q4 (most deprived) | 31849 (24.7) | 11226 (25.9) |  |  | 43041.6 (25.0) | 42693.9 (24.9) |  |
| Education (n, %)* |  |  | 0.076 |  |  |  | 0.025 |
| Secondary qualification | 48673 (37.8) | 17093 (39.4) |  |  | 65562.8 (38.1) | 65526.5 (38.2) |  |
| Post-secondary qualification | 69009 (53.5) | 23380 (53.9) |  |  | 92256.9 (53.6) | 92725.1 (54.1) |  |
| Others | 11226 (8.7) | 2922 (6.7) |  |  | 14384.3 (8.4) | 13139.3 (7.7) |  |
| Lifestyle (n, %)* |  |  | 0.003 |  |  |  | 0.001 |
| Unhealthy | 14975 (11.6) | 5067 (11.7) |  |  | 20137.7 (11.7) | 19532.3 (11.4) |  |
| Moderate | 53607 (41.6) | 17990 (41.5) |  |  | 71538.2 (41.5) | 71234.5 (41.6) |  |
| Healthy | 60326 (46.8) | 20338 (46.9) |  |  | 80528.0 (46.8) | 80624.1 (47.0) |  |
| Common chronic diseases (n, %) |  |  |  |  |  |  |  |
| Hypertension | 42462 (32.9) | 13223 (30.5) | 0.053 |  | 55697.3 (32.3) | 55102.9 (32.2) | 0.004 |
| Cancer | 39921 (31.0) | 13096 (30.2) | 0.017 |  | 52777.8 (30.6) | 53167.6 (31.0) | 0.008 |
| Hyperlipidaemia | 26735 (20.7) | 8425 (19.4) | 0.033 |  | 35056.7 (20.4) | 35210.9 (20.5) | 0.005 |
| Asthma | 17274 (13.4) | 6680 (15.4) | 0.057 |  | 23849.9 (13.8) | 24093.4 (14.1) | 0.006 |
| Depression | 13234 (10.3) | 4706 (10.8) | 0.019 |  | 18020.3 (10.5) | 17600.4 (10.3) | 0.006 |
| Anxiety | 8197 (6.4) | 3008 (6.9) | 0.023 |  | 11141.5 (6.5) | 11176.1 (6.5) | 0.002 |
| T2DM | 6582 (5.1) | 2063 (4.8) | 0.016 |  | 8701.3 (5.1) | 8317.4 (4.9) | 0.009 |
| Autoimmune disease | 6034 (4.7) | 2279 ( 5.3) | 0.026 |  | 8242.7 ( 4.8) | 8586.8 ( 5.0) | 0.010 |
| Chronic kidney disease | 4444 (3.4) | 1337 (3.1) | 0.021 |  | 5788.9 (3.4) | 5687.6 (3.3) | 0.002 |
| Coronary heart disease | 3701 (2.9) | 1143 (2.6) | 0.014 |  | 4861.5 (2.8) | 4703.4 (2.7) | 0.005 |
| COPD | 3425 (2.7) | 1084 (2.5) | 0.010 |  | 4542.9 (2.6) | 4404.6 (2.6) | 0.004 |

SMD, standardized mean difference. * SMD was computed by treating the ordinal variable as if it were continuous.

**Figure 1. Associations of COVID-19 with the presence of 45 symptoms potentially related to PCC during the post-acute phase of infection**


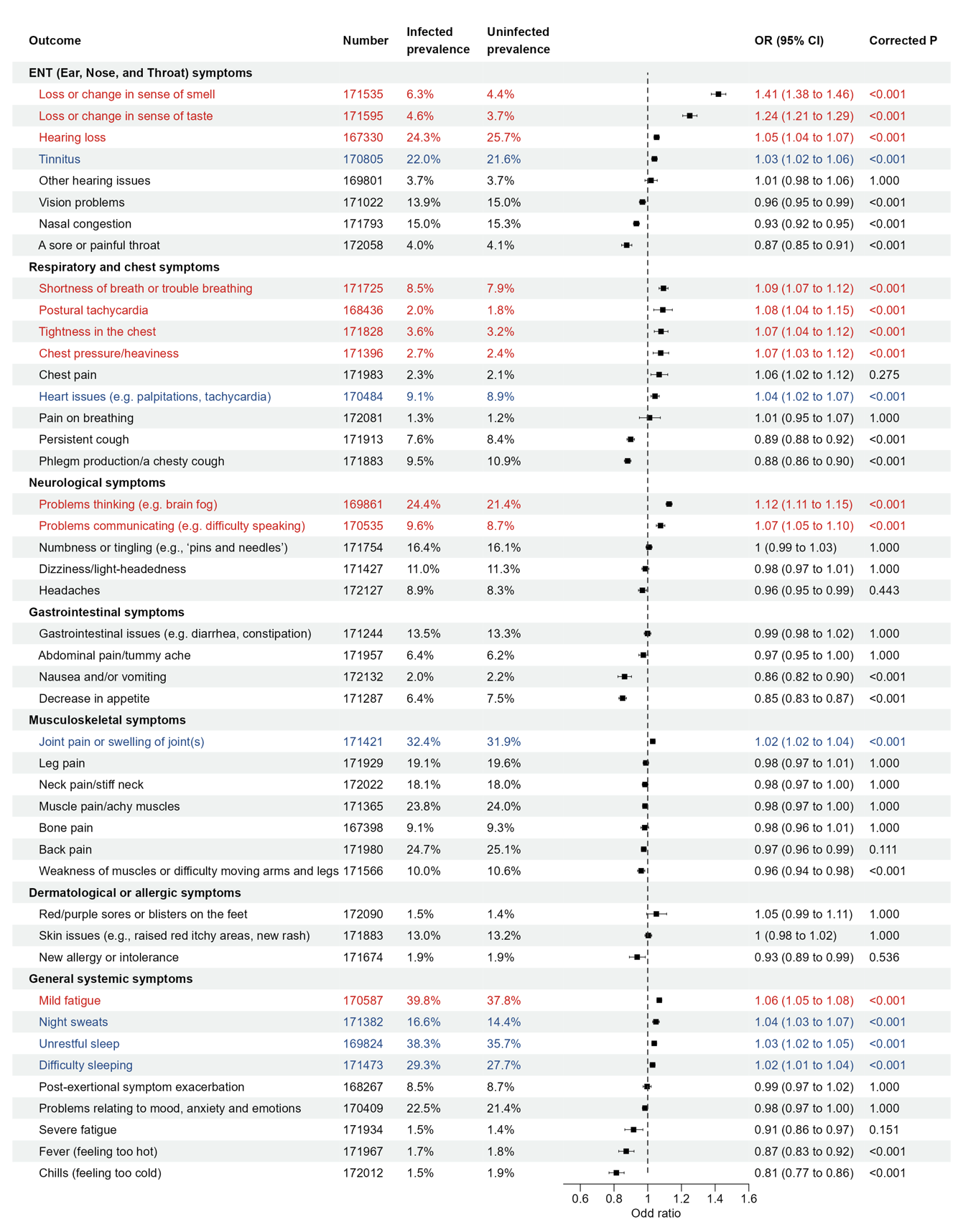


*Unweighted proportion (%) of outcomes in the infected and uninfected group. Number indicates the sample size for each specific symptom. Odds ratios (ORs) were calculated using PS-weighted logistic regression. Symptoms significantly associated with COVID-19 were identified, with red indicating both statistical significance (P < 0.05 after Bonferroni correction) and clinical significance (absolute risk increase >5%), and blue indicating statistical significance but no clinical significance.

**Figure 2. Associations of SARS-CoV-2 pathogen-related factors and vaccination status with PCC and its subtypes.**


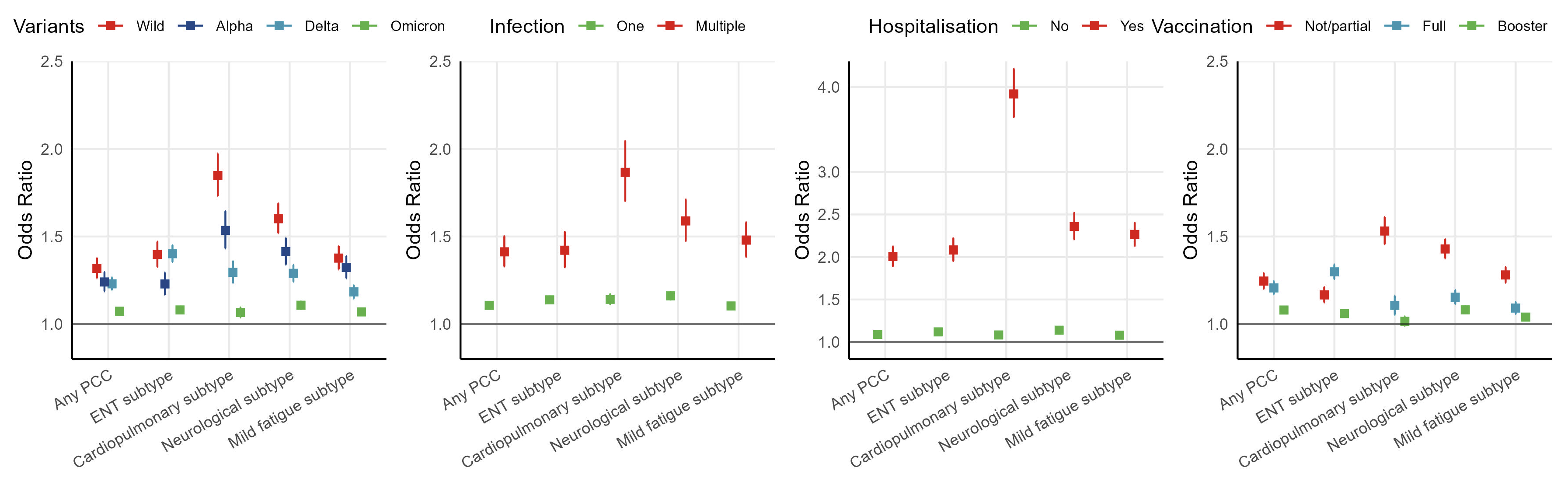


Mean duration between time of infection and time of survey (months): Omicron (4.7), Delta (9.8), Pre-Delta (16.1), and Wildtype (21.3). Hospitalization indicates whether people was admitted to hospital during the acute infection stage (0-30 days). The reference group for all analyses was participants with no evidence of infection.

**Figure 3. Associations of host-related factors with PCC and its subtypes**
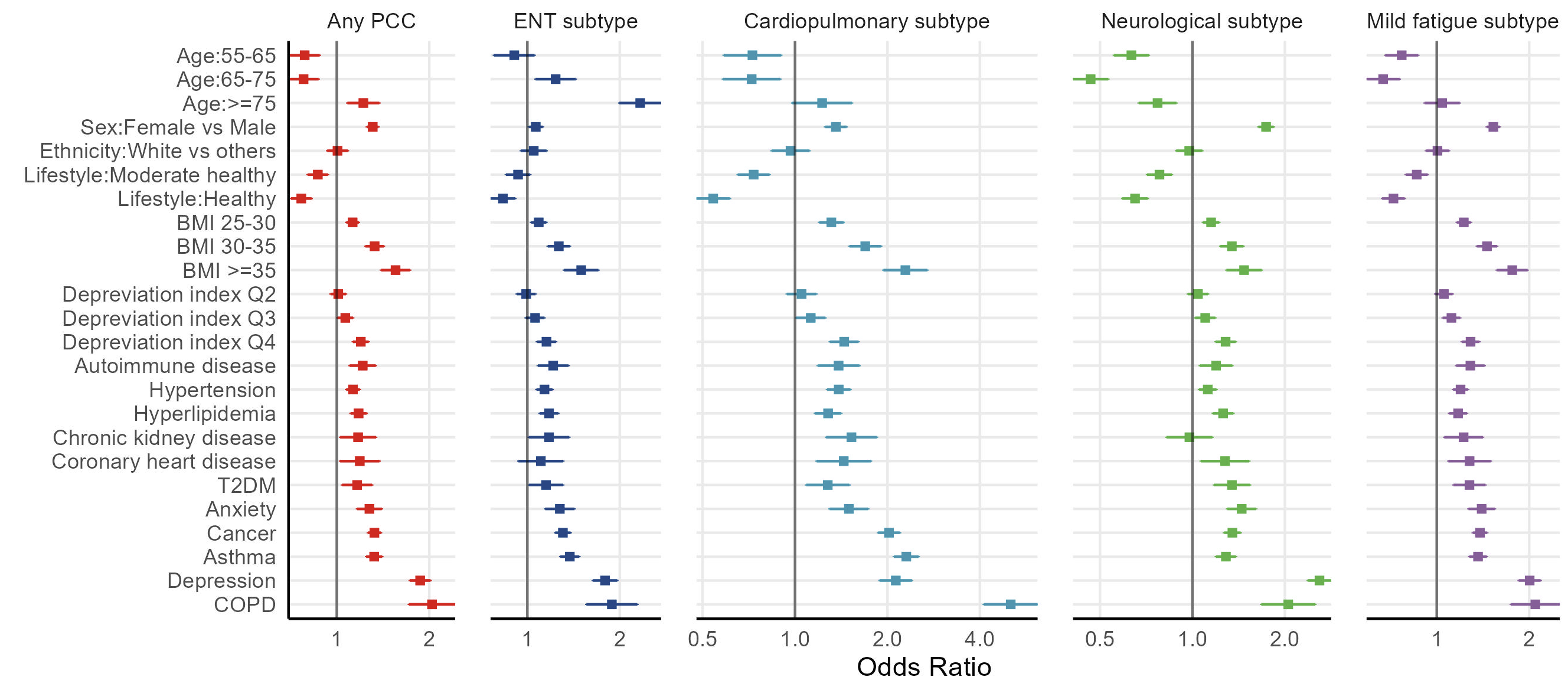


The reference group for age is the 50-55 years, for lifestyle is the unfavorable, for BMI is <25, for deprivation index is Q1, and for the commodities are those not presented. The analyses were conducted among participants with COVID-19.

**Figure 4. Associations of PCC and its subtypes with physical and mental function impairments**


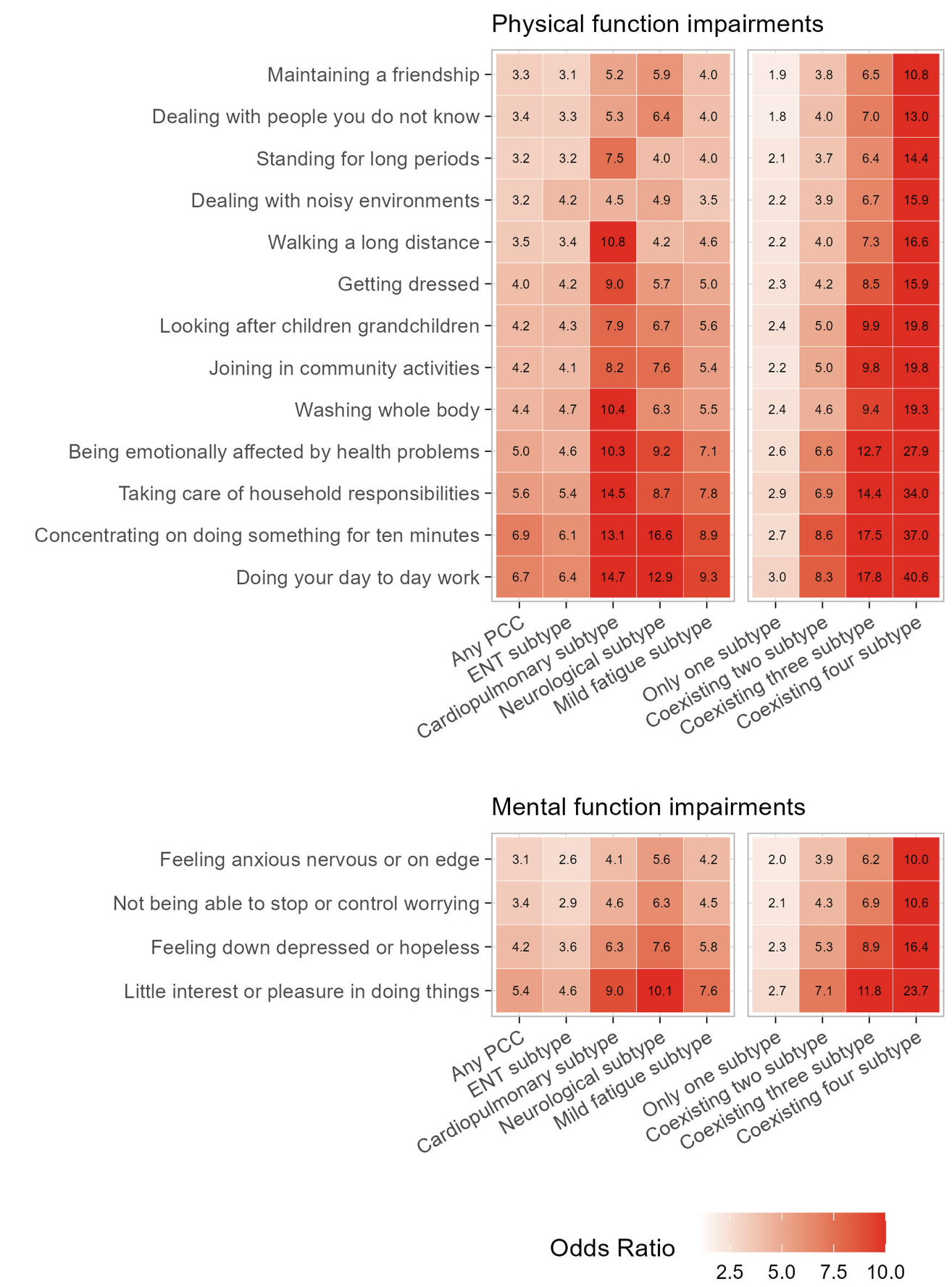


# **Reference**

1. Bowe B, Xie Y, Al-Aly Z. Postacute sequelae of COVID-19 at 2 years. *Nature Medicine* 2023: 1-11.

2. Ballouz T, Menges D, Anagnostopoulos A, et al. Recovery and symptom trajectories up to two years after SARS-CoV-2 infection: population based, longitudinal cohort study. *Bmj* 2023; **381**: e074425.

3. Davis HE, McCorkell L, Vogel JM, Topol EJ. Long COVID: major findings, mechanisms and recommendations. *Nature Reviews Microbiology* 2023; **21**(3): 133-46.

4. Katz GM, Bach K, Bobos P, et al. Understanding How Post–COVID-19 Condition Affects Adults and Health Care Systems. JAMA Health Forum; 2023: American Medical Association; 2023. p. e231933-e.

5. O'Mahoney LL, Routen A, Gillies C, et al. The prevalence and long-term health effects of Long Covid among hospitalised and non-hospitalised populations: A systematic review and meta-analysis. *EClinicalMedicine* 2023; **55**: 101762.

6. Chaichana U, Man KKC, Chen A, et al. Definition of Post-COVID-19 Condition Among Published Research Studies. *JAMA Netw Open* 2023; **6**(4): e235856.

7. Munblit D, Nicholson T, Akrami A, et al. A core outcome set for post-COVID-19 condition in adults for use in clinical practice and research: an international Delphi consensus study. *The Lancet Respiratory Medicine* 2022; **10**(7): 715-24.

8. Munblit D, O'Hara ME, Akrami A, Perego E, Olliaro P, Needham DM. Long COVID: aiming for a consensus. *The Lancet Respiratory Medicine* 2022; **10**(7): 632-4.

9. Zhang HG, Honerlaw JP, Maripuri M, et al. Potential pitfalls in the use of real-world data for studying long COVID. *Nature Medicine* 2023; **29**(5): 1040-3.

10. Canas LS, Molteni E, Deng J, et al. Profiling post-COVID-19 condition across different variants of SARS-CoV-2: a prospective longitudinal study in unvaccinated wild-type, unvaccinated alpha-variant, and vaccinated delta-variant populations. *Lancet Digit Health* 2023; **5**(7): e421-e34.

11. Zhang H, Zang C, Xu Z, et al. Data-driven identification of post-acute SARS-CoV-2 infection subphenotypes. *Nature Medicine* 2023; **29**(1): 226-35.

12. Reese JT, Blau H, Casiraghi E, et al. Generalisable long COVID subtypes: findings from the NIH N3C and RECOVER programmes. *EBioMedicine* 2023; **87**: 104413.

13. Thaweethai T, Jolley SE, Karlson EW, et al. Development of a Definition of Postacute Sequelae of SARS-CoV-2 Infection. *JAMA* 2023; **329**(22): 1934-46.

14. Subramanian A, Nirantharakumar K, Hughes S, et al. Symptoms and risk factors for long COVID in non-hospitalized adults. *Nature Medicine* 2022; **28**(8): 1706-14.

15. Brightling CE, Evans RA, Singapuri A, Smith N, Wain LV. Long COVID research: an update from the PHOSP-COVID Scientific Summit. *The Lancet Respiratory Medicine* 2023; **11**(11): e93-e4.

16. Kuan V, Denaxas S, Gonzalez-Izquierdo A, et al. A chronological map of 308 physical and mental health conditions from 4 million individuals in the English National Health Service. *The Lancet Digital Health* 2019; **1**(2): e63-e77.

17. Denaxas S, Gonzalez-Izquierdo A, Direk K, et al. UK phenomics platform for developing and validating electronic health record phenotypes: CALIBER. *Journal of the American Medical Informatics Association* 2019; **26**(12): 1545-59.

18. Bycroft C, Freeman C, Petkova D, et al. The UK Biobank resource with deep phenotyping and genomic data. *Nature* 2018; **562**(7726): 203-9.

19. Wang Y, Su B, Alcalde-Herraiz M, et al. Modifiable lifestyle factors and the risk of post-COVID-19 multisystem sequelae, hospitalization, and death. *Nat Commun* 2024; **15**(1): 6363.

20. Wang Y, Su B, Xie J, Garcia-Rizo C, Prieto-Alhambra D. Long-term risk of psychiatric disorder and psychotropic prescription after SARS-CoV-2 infection among UK general population. *Nature Human Behaviour* 2024; **8**(6): 1076-87.

21. Bland JM, Altman DG. Multiple significance tests: the Bonferroni method. *BMJ (Clinical Research ed)* 1995; **310**(6973): 170.

22. Ioannidis JPA. The Importance of Predefined Rules and Prespecified Statistical Analyses: Do Not Abandon Significance. *JAMA* 2019; **321**(21): 2067-8.

23. Ballering AV, van Zon SKR, Olde Hartman TC, Rosmalen JGM. Persistence of somatic symptoms after COVID-19 in the Netherlands: an observational cohort study. *Lancet* 2022; **400**(10350): 452-61.

24. Ballouz T, Menges D, Anagnostopoulos A, et al. Recovery and symptom trajectories up to two years after SARS-CoV-2 infection: population based, longitudinal cohort study. *BMJ (Clinical Research ed)* 2023; **381**: e074425.

25. Peter RS, Nieters A, Kräusslich H-G, et al. Post-acute sequelae of covid-19 six to 12 months after infection: population based study. *BMJ (Clinical Research ed)* 2022; **379**: e071050.

26. Atchison CJ, Davies B, Cooper E, et al. Long-term health impacts of COVID-19 among 242,712 adults in England. *Nature Communications* 2023; **14**(1): 6588.

27. Mizrahi B, Sudry T, Flaks-Manov N, et al. Long covid outcomes at one year after mild SARS-CoV-2 infection: nationwide cohort study. *BMJ (Clinical Research ed)* 2023; **380**: e072529.

28. Whitaker M, Elliott J, Chadeau-Hyam M, et al. Persistent COVID-19 symptoms in a community study of 606,434 people in England. *Nature Communications* 2022; **13**(1): 1957.

29. Cooper E, Lound A, Atchison CJ, et al. Awareness and perceptions of Long COVID among people in the REACT programme: Early insights from a pilot interview study. *PLoS One* 2023; **18**(1): e0280943.

30. UK government. COVID-19 Response: Living with COVID-19. 2022. <https://www.gov.uk/government/publications/covid-19-response-living-with-covid-19> (accessed 8/26 2024).

31. Tsampasian V, Elghazaly H, Chattopadhyay R, et al. Risk Factors Associated With Post−COVID-19 Condition: A Systematic Review and Meta-analysis. *JAMA Internal Medicine* 2023; **183**(6): 566-80.

32. UK - Office for National Statistics. Self-reported long COVID symptoms. . <https://www.ons.gov.uk/peoplepopulationandcommunity/healthandsocialcare/conditionsanddiseases/bulletins/selfreportedlongcovidsymptomsuk/10july2023> (accessed November 15, 2023.

33. Magnusson K, Kristoffersen DT, Dell'Isola A, et al. Post-covid medical complaints following infection with SARS-CoV-2 Omicron vs Delta variants. *Nature Communications* 2022; **13**(1): 7363.

34. Antonelli M, Pujol JC, Spector TD, Ourselin S, Steves CJ. Risk of long COVID associated with delta versus omicron variants of SARS-CoV-2. *Lancet* 2022; **399**(10343): 2263-4.

35. Tsampasian V, Elghazaly H, Chattopadhyay R, et al. Risk factors associated with Post− COVID-19 condition: a systematic review and meta-analysis. *JAMA Internal Medicine* 2023.

36. Bowe B, Xie Y, Al-Aly Z. Acute and postacute sequelae associated with SARS-CoV-2 reinfection. *Nature Medicine* 2022; **28**(11): 2398-405.

37. Català M, Mercadé-Besora N, Kolde R, et al. The effectiveness of COVID-19 vaccines to prevent long COVID symptoms: staggered cohort study of data from the UK, Spain, and Estonia. *The Lancet Respiratory Medicine* 2024; **12**(3): 225-36.

38. Taquet M, Sillett R, Zhu L, et al. Neurological and psychiatric risk trajectories after SARS-CoV-2 infection: an analysis of 2-year retrospective cohort studies including 1 284 437 patients. *Lancet Psychiatry* 2022; **9**(10): 815-27.

39. Thompson RC, Simons NW, Wilkins L, et al. Molecular states during acute COVID-19 reveal distinct etiologies of long-term sequelae. *Nature Medicine* 2023; **29**(1): 236-46.

40. Liew F, Efstathiou C, Fontanella S, et al. Large scale phenotyping of long COVID inflammation reveals mechanistic subtypes of disease. *medRxiv* 2023: 2023.06.07.23291077.

41. Batty GD, Gale CR, Kivimäki M, Deary IJ, Bell S. Comparison of risk factor associations in UK Biobank against representative, general population based studies with conventional response rates: prospective cohort study and individual participant meta-analysis. *BMJ (Clinical Research ed)* 2020; **368**: m131.

**Supplemental Online Content**

**Refinement of post-COVID condition subtypes, determinants, and health impacts**

|  |
| --- |
| **Supplementary Figures** |
| Supplementary Figure 1. Study design and the process of cohort construction |
| Supplementary Figure 2. Correlation patterns of the 45 PCC-related symptoms in 7 domains |
| Supplementary Figure 3. Distribution of index dates for the infection and control cohorts over the study period |
| Supplementary Figure 4. Prevalence of multiple chronic diseases in participants with and without PCC |
| Supplementary Figure 5. Associations of COVID-19 with 45 PCC-related symptoms during the post-acute phase of infection using other definitions |
| Supplementary Figure 6. Prevalence of 45 PCC-related symptoms in participants with and without COVID-19 by definition |
| **Supplementary Tables** |
| Supplementary Table 1. Origins and sources of questionnaire items used in the UK Biobank online Health and Well-being survey |

**Supplementary Figure 1.** **Study design and the process of cohort construction.**

172,303

Participants completed health and well-being questionnaire included

333,427*

Participants with valid email address invited from ~400,000 UKB participants alive

195,680

Participants completed health and well-being questionnaire as of May 18, 2022

1,094

Participants completed the questionnaire by participant website

23,357

Participants who completed the survey after the end data of data linkage (Sept 30, 2022) or had COVID-19 within 30 days prior to the survey

43,395

Infected group

128,908

Uninfected group

Propensity score weighting

Identification of most indicative PCC symptomatology from 45 predefined PCC-related symptoms

Refinement of PCC subtypes based on identified symptomatology and prior evidence in clinical practice and research

Identification of the pathogen-related and host-related determinants of PCC and subtypes

Identification of the physical and mental function impairments of PCC and subtypes

Pathogen-related:

Infected group vs. uninfected group

Host-related:

PCC group vs. non-PCC group among infected group

PCC group vs. non-PCC group among infected group

Uninfected group

Assigned T_0_

Infected group

T_0_

T_0_ +30 days

Reported PCC-related symptoms in the survey

**Supplementary Figure 2.** **Correlation patterns of the 45 PCC-related symptoms in 7 domains.**


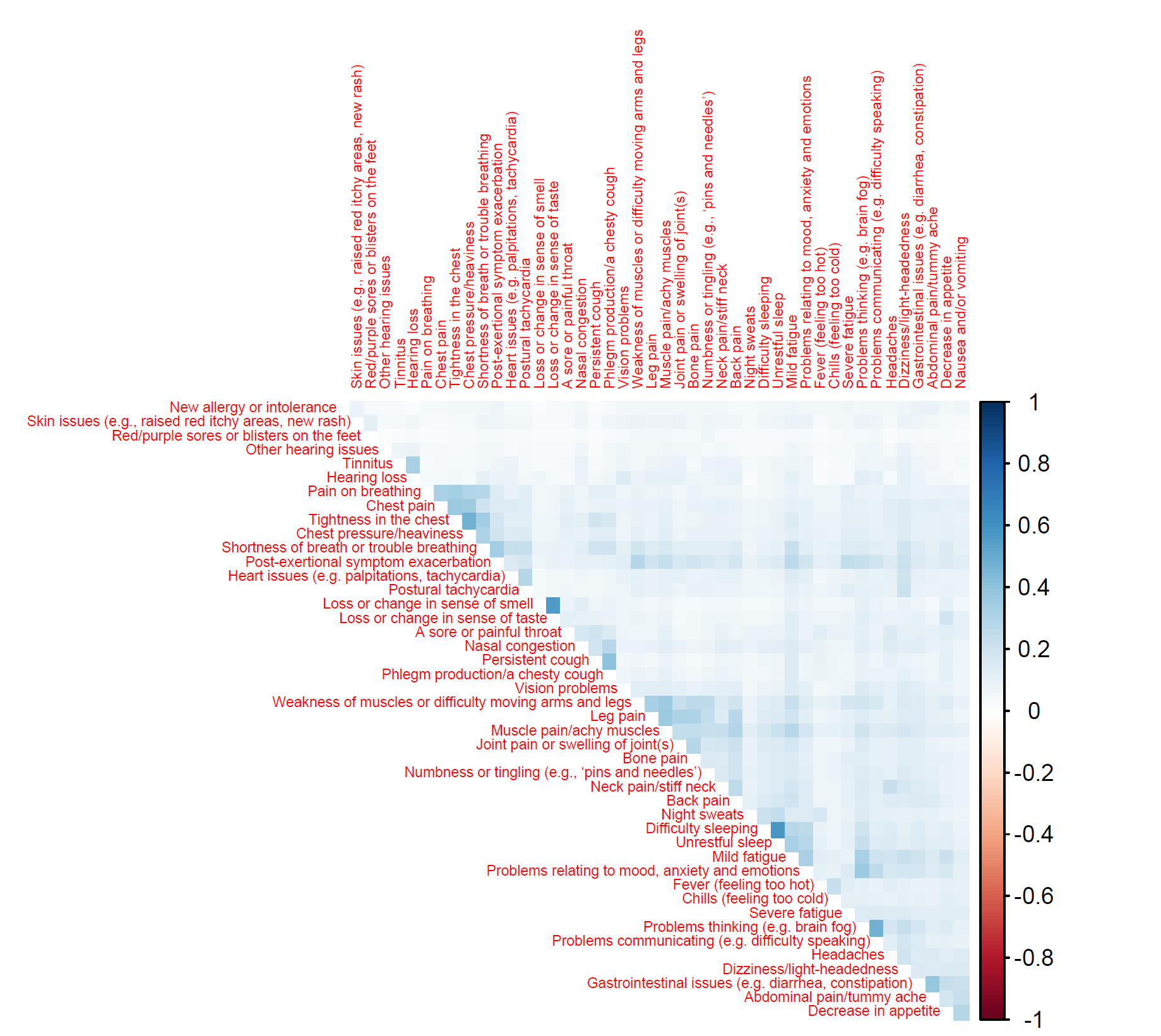


**Supplementary Figure 3.** **Distribution of index dates for the infection and control cohorts over the study period.**


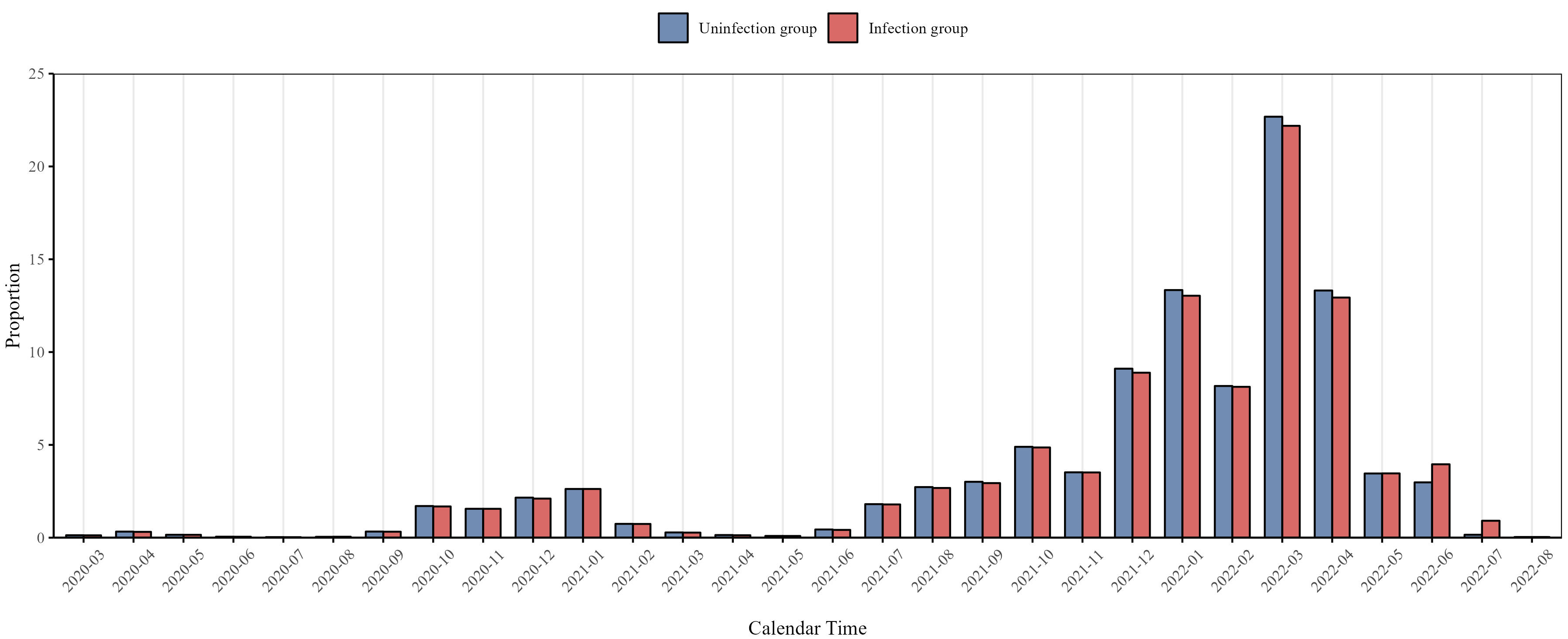


**Supplementary Figure 4. Prevalence of multiple chronic diseases in participants with and without PCC**

**Supplementary Figure 5. Associations of COVID-19 with 45 PCC-related symptoms during the post-acute phase of infection using other definitions**

**a**, presence of symptoms 90 days beyond infection **b**, presence of symptoms 90 days beyond infection and lasting ≥4weeks


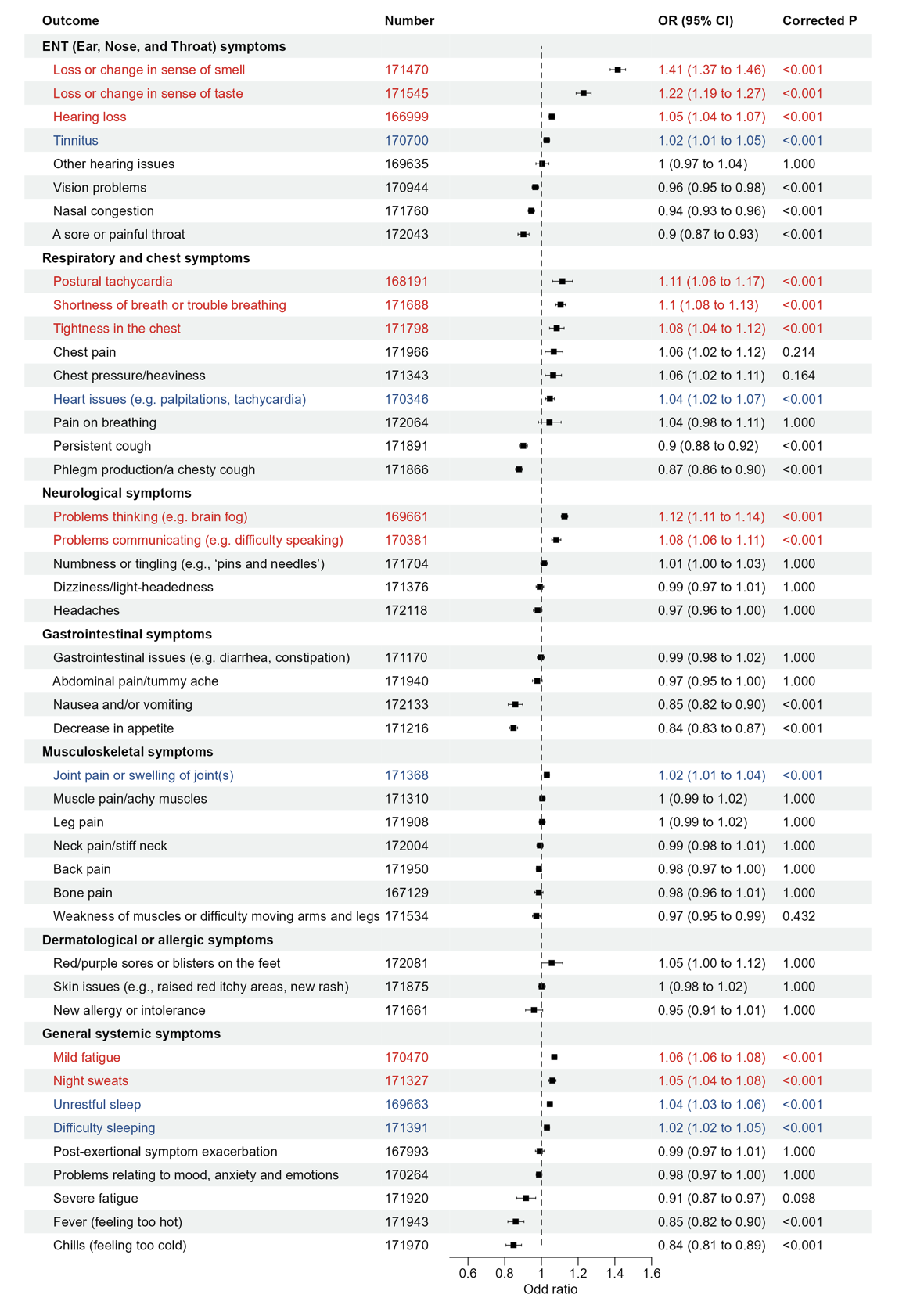
**
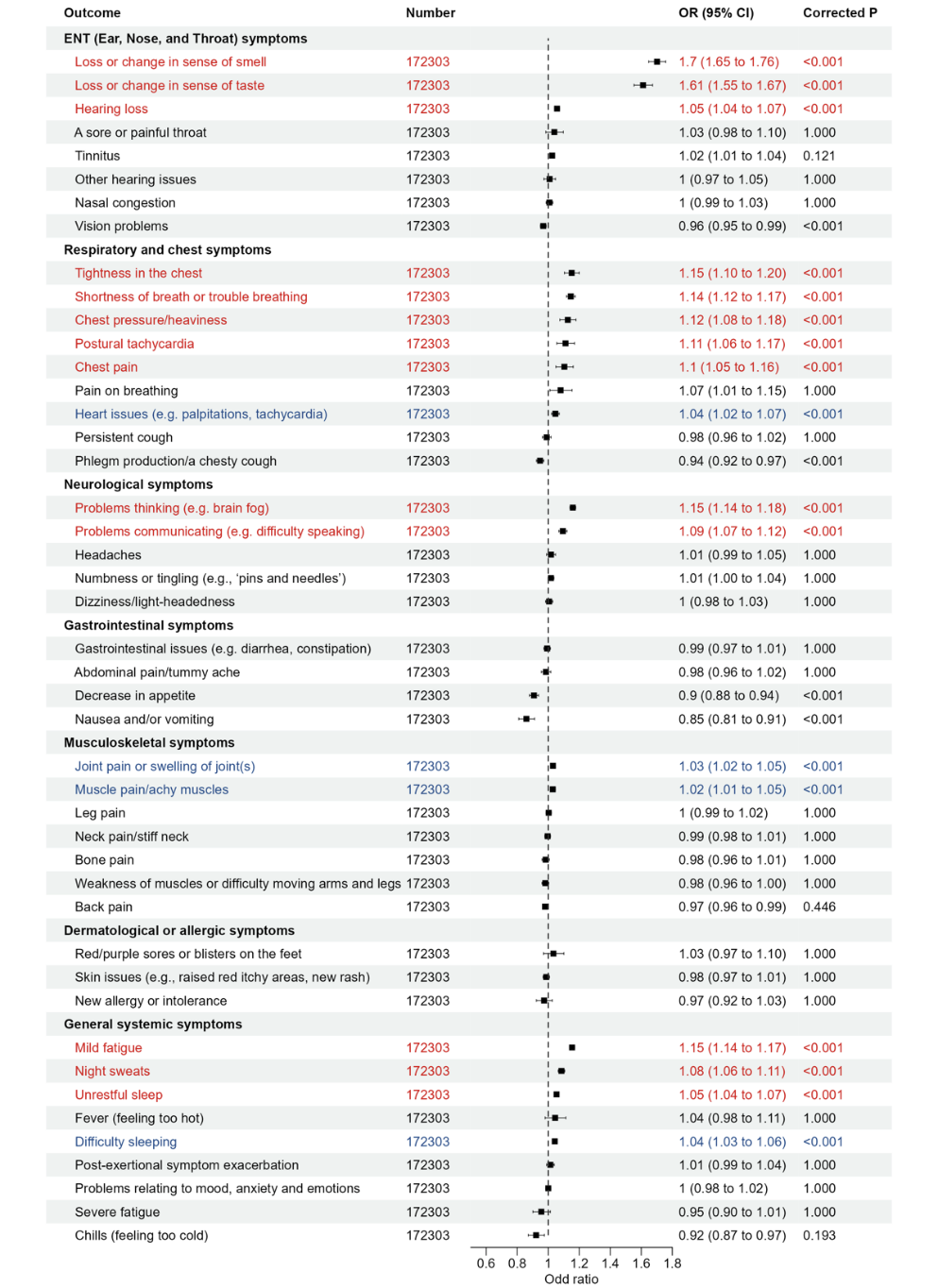
**

**Supplementary Figure 6. Prevalence of 45 PCC-related symptoms in participants with and without COVID-19 by definition**

**
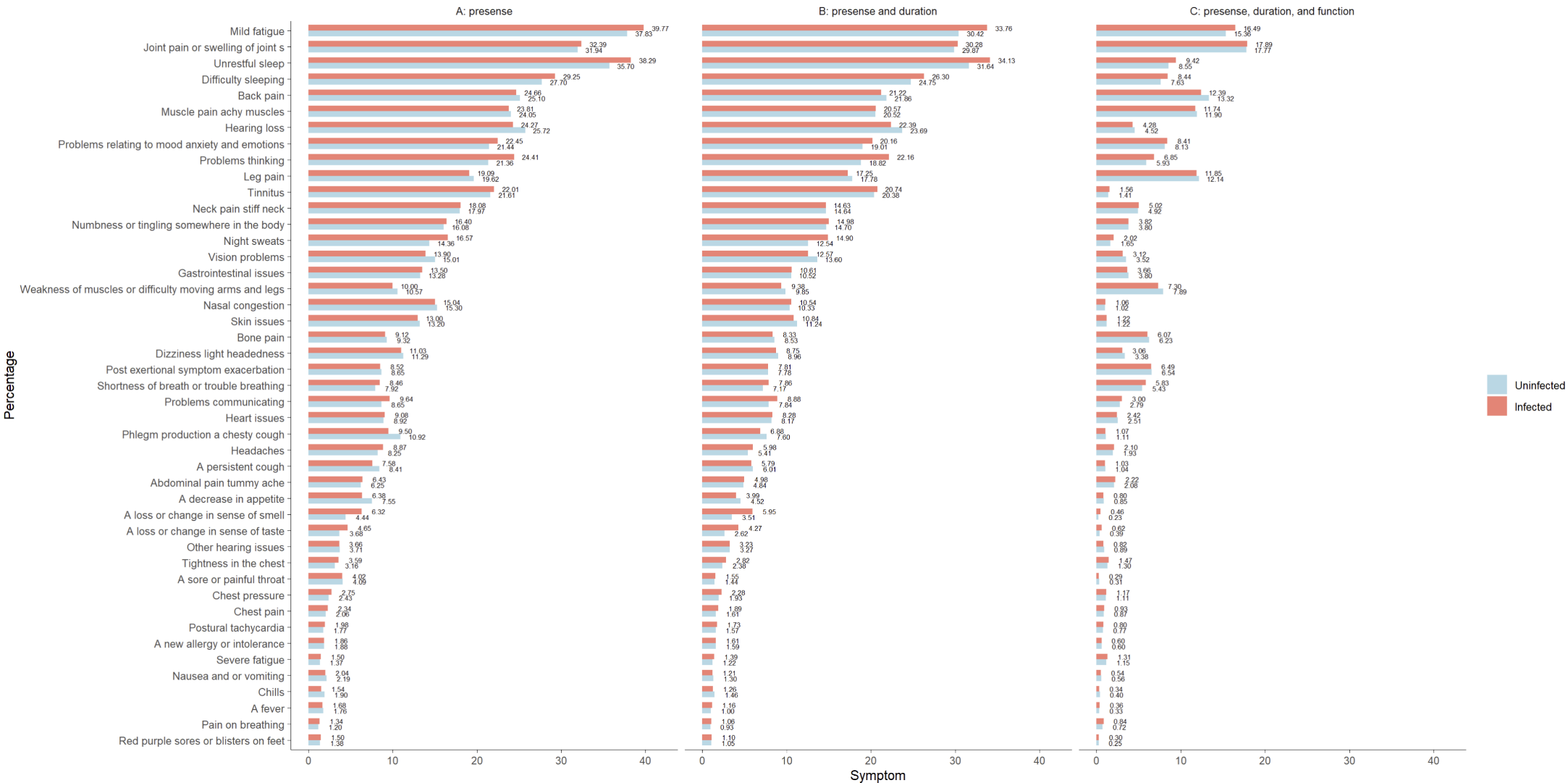
**

A, presence of symptom during post-acute phase of infection (30 days beyond acute infection)

B, presence of symptom during post-acute phase of infection (30 days beyond acute infection) that lasted more than 4 weeks

C, presence of symptom during post-acute phase of infection (30 days beyond acute infection) that lasted more than 4 weeks and cause participants to avoid, reduce or spread out their usual activities

**Supplementary Table 1. Origins and sources of questionnaire items used in the UK Biobank online Health and Well-being survey**

| **Q. ID** | **Source** | **Authors** |  | **Available at** |
| --- | --- | --- | --- | --- |
| HT1 | Q1.2.1 and Q2.2.1 of Wellcome Trust Longitudinal Population Studies (LPS) Questionnaire Resource v0.5, June 2021 | Questionnaire provided by Wellcome Longitudinal Population Study COVID-  19 Steering Group and Secretariat (221574/Z/20/Z) |  | [https://www.bristol.ac.uk/alspac/covid-](https://www.bristol.ac.uk/alspac/covid-19/wellcome-covid-19/) [19/wellcome-covid-19/](https://www.bristol.ac.uk/alspac/covid-19/wellcome-covid-19/) |
|  | TwinsUK COVID-19 Health and Personal Experiences (CoPE) Questionnaire, v55 | Department of Twin Research & Genetic Epidemiology, King’s College London |  | From TwinsUK study team |
|  | Office for National Statistics (ONS) COVID-19 infection survey of the UK general population | Office for National Statistics and the University of Oxford (UK), delivered by  IQVIA (USA) |  | [https://www.ndm.ox.ac.uk/covid-19/covid-](https://www.ndm.ox.ac.uk/covid-19/covid-19-infection-survey/case-record-forms) [19-infection-survey/case-record-forms](https://www.ndm.ox.ac.uk/covid-19/covid-19-infection-survey/case-record-forms) |
|  | World Health Organization (WHO) Global COVID-19 Clinical Platform Case Report Form (CRF) for Post COVID Condition (Post COVID-19 CRF) | Health Care Readiness Unit, WHO |  | [https://www.who.int/publications/i/item/glob](https://www.who.int/publications/i/item/global-covid-19-clinical-platform-case-report-form-(crf)-for-post-covid-conditions-(post-covid-19-crf-)) [al-covid-19-clinical-platform-case-report-](https://www.who.int/publications/i/item/global-covid-19-clinical-platform-case-report-form-(crf)-for-post-covid-conditions-(post-covid-19-crf-)) [form-(crf)-for-post-covid-conditions-(post-](https://www.who.int/publications/i/item/global-covid-19-clinical-platform-case-report-form-(crf)-for-post-covid-conditions-(post-covid-19-crf-)) [covid-19-crf-)](https://www.who.int/publications/i/item/global-covid-19-clinical-platform-case-report-form-(crf)-for-post-covid-conditions-(post-covid-19-crf-)) |
|  | Prof Matthew Hotopf and Dr Katrina Davis’ work on long COVID | Developed in conjunction with the UK Biobank Mental Health Outcomes Consortium and external experts on long COVID and outcome measures.  Submitted to UK Biobank’s Strategic Oversight Committee in support of application to undertake second mental health questionnaire (now relabeled as  mental well-being questionnaire, 2022) |  | Internal document |
|  | UK Biobank mental well-being questionnaire 2022 | Developed in conjunction with Prof Matthew Hotopf and Dr Katrina Davis at KCL, in collaboration with UKB Mental Health Outcomes Consortium.  Given favourable ethical opinion by North West - Haydock REC, amendment 01, August 2021 |  | Questionnaire developed for online use via the University of Oxford’s Online Questionnaire Sharing (OQS) Service |
|  | COVID-19 rapid guideline: managing the long-term effects of COVID-19 | NICE guideline covering the identification, assessment and management of the long-term effects of COVID-19 |  | <https://www.nice.org.uk/guidance/ng188> |

|  | BMJ Best Practice: Coronavirus disease 2019 (COVID-  19) | BMJ Best Practice Guidance provided  for medical professionals |  | [https://bestpractice.bmj.com/topics/en-](https://bestpractice.bmj.com/topics/en-gb/3000201/complications)  [gb/3000201/complications](https://bestpractice.bmj.com/topics/en-gb/3000201/complications) |
| --- | --- | --- | --- | --- |
|  | Real-time Assessment of Community Transmission Long COVID (REACT-LC) survey 2022 | Faculty of Medicine, Imperial College London |  | [https://www.imperial.ac.uk/medicine/resear](https://www.imperial.ac.uk/medicine/research-and-impact/groups/react-study/studies/react-long-covid/) [ch-and-impact/groups/react-](https://www.imperial.ac.uk/medicine/research-and-impact/groups/react-study/studies/react-long-covid/) [study/studies/react-long-covid/](https://www.imperial.ac.uk/medicine/research-and-impact/groups/react-study/studies/react-long-covid/) |
| HT2 | Developed by UK Biobank |  |  |  |
| HT3 | Developed by UK Biobank, using response options taken  from UK Biobank mental well-being questionnaire work with Hotopf/Davis |  |  |  |
| PHQ 4 | Patient Health Questionnaire for Depression and Anxiety (PHQ-4) | Developed by Drs. Robert L. Spitzer, Janet B.W. Williams, Kurt Kroenke and colleagues, with an educational grant from Pfizer Inc. No permission required to reproduce, translate, display or  distribute |  | [https://www.phqscreeners.com/select-](https://www.phqscreeners.com/select-screener) [screener](https://www.phqscreeners.com/select-screener) |
| EL1 | Amended version of Q2.2.15 of Wellcome Trust LPS Questionnaire Resource v0.5, June 2021 (response options amended and presented in a different format) | Questionnaire provided by Wellcome Longitudinal Population Study COVID- 19 Steering Group and Secretariat  (221574/Z/20/Z) |  | [https://www.bristol.ac.uk/alspac/covid-](https://www.bristol.ac.uk/alspac/covid-19/wellcome-covid-19/) [19/wellcome-covid-19/](https://www.bristol.ac.uk/alspac/covid-19/wellcome-covid-19/) |
| EL2 | Q2.2.16 of Wellcome Trust LPS Questionnaire Resource v0.5, June 2021 (responses presented in a different order) | Questionnaire provided by Wellcome Longitudinal Population Study COVID-  19 Steering Group and Secretariat (221574/Z/20/Z) |  | [https://www.bristol.ac.uk/alspac/covid-](https://www.bristol.ac.uk/alspac/covid-19/wellcome-covid-19/) [19/wellcome-covid-19/](https://www.bristol.ac.uk/alspac/covid-19/wellcome-covid-19/) |
| C1 | Developed by UK Biobank |  |  |  |

The full list of questions can be found in the online resource for online Health and Well-being survey (<https://biobank.ctsu.ox.ac.uk/crystal/refer.cgi?id=2500>).
